# Improving the Radical Cure of *Plasmodium vivax* Malaria

**DOI:** 10.4269/ajtmh.14-0118

**Published:** 2014-07-02

**Authors:** Ric N. Price

**Affiliations:** Centre for Tropical Medicine, Nuffield Department of Clinical Medicine, University of Oxford, Oxford, United Kingdom; Global Health Division, Menzies School of Health Research, Charles Darwin University, Casuarina, Darwin, Australia

Outside of Africa, *Plasmodium falciparum* almost invariably coexists with *P. vivax*. The biology of these two parasites is notably different, rendering *P. vivax* more resilient to conventional malaria control measures. In coendemic areas, the successful reduction in the burden of *P. falciparum* often leaves *P. vivax* as the main cause of malaria, significantly undermining our aspirations for the elimination of malaria. Unlike *P. falciparum*, *P. vivax* infections form dormant liver stages (hypnozoites), which can cause relapses of infection weeks to months after the initial attack.^1^ Repeated relapses damage the health and development of patients, particularly young children, resulting in chronic and severe anemia, malnutrition, growth retardation, and poor school attendance. Regarded at one time as a benign infection, vivax malaria is now recognized as a major cause of morbidity and an important contributor to mortality.^2^ The latter is well-illustrated in this edition of the *American Journal of Tropical Medicine and Hygiene* in the work by Quispe and others,^3^ which is from an observational study conducted in Peru, and the accompanying editorial by Baird.^4^

One of the greatest challenges in *P. vivax* therapeutics is how to achieve radical cure safely and reliably. Radical cure requires delivery of antimalarial agents targeting both the erythrocytic stages of infection as well as the liver stages. The only licensed antimalarial with hypnozoitocidal activity is primaquine, an 8-aminoquinoline drug that can also cause severe hemolysis in patients with glucose-6-phosphate dehydrogenase (G6PD) deficiency, an enzyme deficiency present in 1–40% of the population.^5^ Current World Health Organization (WHO) guidelines recommend a 14-day primaquine regimen primarily for safety concerns, and therefore, the drug can be stopped if significant hemolysis occurs. However, because primaquine treatment is usually unsupervised, adherence to such a prolonged course of antimalarials is generally poor, limiting its effectiveness and public health benefit.

The main determinant of primaquine efficacy is the total dose of primaquine administered rather than the duration over which it is delivered.^6^ Shorter courses with a higher daily dose of primaquine have the potential to improve adherence and thus, effectiveness without compromising efficacy. In Thailand, directly observed primaquine administered over 7 days rather than the same dose over 14 days was well-tolerated and reduced recurrent infections by day 28 to 4%.^7^ This finding is encouraging, but because many relapses present after 1 month, longer follow-up is needed to distinguish whether relapse was prevented or deferred.

In this edition of the *American Journal of Tropical Medicine and Hygiene*, Durand and others^8^ present an important open-label randomized trial of 540 adults and children with symptomatic vivax malaria from Loreto in Peru. The clinical study compared three primaquine treatment regimens for G6PD normal patients with vivax malaria: 0.5 mg/kg per day for 5 days, 0.5 mg/kg per day for 7 days, and 0.25 mg/kg per day for 14 days. All patients received a 3-day regimen of chloroquine. After 6 months of follow-up, the risk of recurrence in the 5-day treatment arm was high (28%), which was in keeping with previous observations that a total dose of 2.5 mg/kg is insufficient to prevent *P. vivax* relapses.^6^ However, the risk of recurrence was considerably lower in patients taking either the 7- (10.2%) or the 14-day regimen (13.5%). The difference in efficacy between the 7- and 14-day regimens was not significant. Reassuringly, the 7-day regimen was well-tolerated, with no moderate or severe adverse effects reported, although no data on hemoglobin concentrations were provided.

As acknowledged by Durand and others,^8^ a total dose of 3.5 mg/kg primaquine administered in the 7- and 14-day regimens seems to be at the edge of reasonable efficacy. Some of the recurrences observed in the study by Durand and others^8^ were likely reinfections rather than relapses; however, the survival curves presented suggest that the rate of reinfection was low, because after 120 days, the rate of recurrence had fallen off in the 7- and 14-day treatment arms. Other studies also suggest that, although a 3.5-mg/kg total dose regimen has reasonable efficacy in some locations, but in many settings it is associated with high rates of treatment failure. Moreover, this regimen is more vulnerable to patients failing to adhere to a complete course of treatment.^6^ In a study from Brazilian Amazonia, a similar total dose of primaquine administered over 7 days was associated with 26–40% recurrences at 6 months.^9^ The most recent WHO guidelines now recommend a higher dose of primaquine, with a total dose of 7 mg/kg, but additional studies will be needed to assess the minimum number of days over which this dosing regimen could be safely administered.^10^

The WHO therapeutic guidelines recommend that patients should be tested for G6PD deficiency before prescribing primaquine. However, diagnostic tests will never be 100% reliable and in practice, are usually unavailable in resource-poor settings. The risk of primaquine-induced hemolysis is dose-dependent; hence, the consequences of inadvertently administering a high-dose primaquine regimen to a G6PD-deficient individual erroneously diagnosed as G6PD normal will need to be gauged before wider recommendations can be made.

After more than half a century of being reliant on primaquine for delivering *P. vivax* radical cure, the prospect of new treatment options is becoming reality. Tafenoquine, currently in phase III clinical trials, has potential to clear the liver of *P. vivax* hypnozoites with a single 300-mg dose.^11^ However, tafenoquine is also an 8-aminoquinoline antimalarial with potential to cause severe hemolysis. Although adherence to a complete course of treatment will undoubtedly be much improved with tafenoquine, the challenges of ensuring patient safety may be even more challenging than with primaquine, because tafenoquine has a long half-life, which will result in patients being exposed to the drug for a prolonged period. Priority now needs to be given to developing robust and affordable G6PD deficiency point-of-care tests, generating greater knowledge of the prevalence of different variants of G6PD deficiency and their associated risks of hemolysis, and improving adherence to a complete course of treatment. Information on risks needs to be balanced against a greater awareness that failing to deliver radical cure comes with its own inherent dangers of recurrent bouts of malaria.^12^ The important paper by Durand and others^8^ in this edition of the *American Journal of Tropical Medicine and Hygiene* highlights that, after years of relative neglect, we are finally making progress in delivering safe and effective antimalarial treatment strategies for *P. vivax*.
